# Clinical Contributions to Obstetrics and Gynecology

**Published:** 1886-03

**Authors:** Joseph A. Eve, Joseph Eve Allen

**Affiliations:** Augusta, Ga.; Augusta, Ga.


VOL. III. MARCH, 1886. No. 1.

### Original Communications.

### CLINICAL CONTRIBUTIONS TO OBSTETRICS AND GYNECOLOGY.

BY JOSEPH A. EVE, M. D., LL. D., AUGUSTA, GA., AND JOSEPH EVE ALLEN, M. D., AUGUSTA, GA.

In the last half century many and great changes have taken place in practical medicine; grand discoveries have been made, new remedies have been introduced, ingenious instruments for precision in diagnosis and facility in operating have been devised, brilliant ideas have been advanced, and startling methods of treatment advocated and established. During this time much that was novel has grown old, much that was old has been proven good, and many things that were new have been abandoned as worthless, numerous highly praised remedies have been shown to be of no value, and fine spun theories have been demonstrated to be fallacious, errors have had their day, wild vagaries have for a season taken hold of the professional mind, and many much-lauded improvements in practice have either been carried to excess or failed to stand the test of experience. Time, the mighty iconoclast, has cast down the older authorities, Sydenham, Broussais, Cooper and Denman, and has raised up in their stead Virchow, Zeimssen, Gross and Barnes. Progress has, however, truly been the spirit of the age, for great and wonderful has been the advancement made in every department of medical science.

Dr. Thomas Denman, in his work on Obstetrics,* congratulated the practitioners of his day on the improvement in the practice of midwifery "within the last sixty or seventy years;" but what is the accoucheur of that time compared with his successor of the present, who, possessed of fuller knowledge and armed with better instruments, more correct methods, and above all having the blessed, the God-given, gift of anæsthesia has resources at his command which were not even dreamed of in the past. Now by chloroform childbirth is robbed of much of its pain, and by the proper employment of antisepsis childbed is rid of many of its perils. Modern research has greatly reduced the rate of mortality among lying-in women, and infants now are saved that formerly were sacrificed. Gynecology, thanks to the intellect of Marion Sims, has grown from the merest empiricism into the first rank among the sciences.

Although in medicine much already has been done, there yet remains much more to be accomplished; there are yet many facts to be found out, many problems to solve, many questions to answer. Nature has yet many secrets to disclose and many hidden laws to be revealed. The vast region of the unknown stretches far beyond us and contains many bright discoveries to immortalize the names of future fortunate investigators. What a grand and glorious object is presented to the ambitious student of to-day! the hope that through his genius and industry something may be added to the stock of human knowledge, something taken from the sum of human woes. Truth in medicine, as in the other sciences, can only be ascertained by long, patient, arduous search. True progress can only be made and proper principles of treatment established by correct and rational deductions and generalizations derived from and based upon a thorough study and comparison of a great number of clinical facts

*An Introduction to the Practice of Midwifery.

and phenomena. It becomes, therefore, the duty of every one who has enjoyed especial opportunities for observation to put his experience on record in order that material may be furnished to the medical philosopher from which to draw conclusions that will enrich science and benefit suffering humanity.

With the view of contributing in some slight degree toward the elucidation of certain questions about which the opinion of the profession is at present divided, the following papers were prepared presenting the results of a life-work in the special departments of obstetrics and gynecology, together with such notes and references as are pertinent to the subject.

### THE MANAGEMENT OF NATURAL LABOR.

Normal labor being a natural physiological process in its management, few and simple are the duties that ordinarily devolve upon the accoucheur and interference on his part is seldom necessary. It is his office to superintend and direct, to foresee dangers, to prevent accidents, and promptly meet emergencies.

In the ability to render aid and assistance when needed to women in this the most trying physical ordeal of their lives consists the dignity and beneficence of the profession of the obstetrician. This it is that raises it far above the vulgar calling of the midwife, makes it worthy of the attention and pursuit of intelligent men, and constitutes it an important element in civilized society.

Patience, a virtue which seems to have no place in this restless, bustling age, is one of the first and most essential qualifications of the obstetrician. Truly there is no wiser maxim in practical medicine to-day than that of the learned Dr. Blundell, of London, "meddlesome midwifery is bad." Such is the great conservatism, the wonderful mechanism, and the beautiful adaptation of means to ends in nature that often results are accomplished by her unaided powers that at first sight appear to be impossible, and rash and untimely interference is always attended with danger and frequently with fearful disaster. Far be it from us to council one moment's delay, when for the sake of either mother or infant action is demanded, but it is our purpose here to point out what in our experience are the indications for and the best ways of giving assistance when during labor it is required.

The first stage of labor in many instances may be long protracted, lasting for a number of hours or even days without any harm resulting to either mother or infant. We have often seen cases, as has every other practitioner of obstetrics, wherein labor has commenced and the uterine contractions progressed vigorously until from some emotional or other cause the pains have stopped and all action for a time completely suspended.* This condition of rest or inertia occurring at this time does not, as in the second stage, call for treatment, because it is salutary in its effects, enabling the system to recuperate its powers and better fitting it for effort during the period of expulsion. Atony or exhaustion of the uterus is, however, an entirely different matter from inertia, and when present or threatened during labor urgently demands the most prompt attention of the medical attendant. When, therefore, the first stage of labor lasts beyond physiological limits assistance is certainly required to finish the delivery, for after long-continued, ineffectual exertion the uterine muscle, like every other muscle of the body, falls first into a state of tonic or tetanic contraction, and then becomes relaxed and paralyzed, the first condition may cause rupture of the womb or asphyxia of the child from obstruction of the placental circulation, and the other predisposes to *post partem* hemorrhage. Again other serious and hurtful consequences may follow abnormal duration of the first stage, such as congestion or inflammation of the cervix and lower portion of the body of the uterus from the constant pressure of the head against these parts, or the woman's strength may break down from the persistence and severity of the pains preventing rest and sleep, and she be unable to make the necessary muscular effort in the second stage or resist the dangers of the puerperal period.

---

*A remarkable case of this kind, showing the powerful controlling influence of strong mental emotions upon the progress of labor, occurred in the practice of the late Dr. Milton Antony. He was once called to attend a patient in the first stage of labor and found the membranes protruding, the os dilating and everything promising a quick and easy delivery, when the woman's husband came home beastly drunk, and her distress and mortification at this so affected the action of the uterus that the labor was entirely arrested and continued so until the debauch ended, a period of a week or ten days.

The proper treatment of delay in the first stage of labor varies with different cases, as it of course depends upon the causes that give rise to the difficulty.

*Artificial Rupture of the Membranes.*—Certain of the older obstetric authorities held that no harm could result so long as the amniotic sack continued intact, and that its artificial rupture was never necessary, no matter how long it remained unbroken. Cases often occur, however, in which the labor has been retarded many hours and the woman brought almost into a state of exhaustion, because from excessive toughness of the membranes or the existence of hydramnion the advance of the head is obstructed and efficient uterine contractions prevented. Such cases usually terminate rapidly and satisfactorily when the amniotic fluid is let off by the accoucheur. The best time for rupturing the bag of waters is when the os is dilated or dilatable and the head can be felt through the somewhat flaccid membranes; it should be done as the pain is passing off, for then the presenting part of the child is forced down into the cervix and thus prolapse of the cord is prevented.

*The Use of Opium.*—Much good can be accomplished by the administration of a full dose of opium in those cases of delay in the first stage of labor, in which the pains are severe but irregular and ineffective, and after hours of effort the cervix still remains unobliterated, and the internal os undilated. In such cases generally the beneficial effect of this drug is to stop the pains, produce sleep, and allow the nervous system of the woman which has become depressed by the prolonged suffering time to recruit its failing powers. When the action of the opium ceases the patient awakes refreshed and invigorated, uterine contraction commences with renewed energy, and the delivery soon follows. Dr. Churchill* advised that after giving an opiate to a parturient woman, the practitioner should never leave her house lest the baby be born in his absence ; for sometimes after its use, instead of the labor being arrested, the parts become relaxed, and the pains increase in force and efficiency. This apparent oxytocic property of opium in certain cases is ex-

*Theory and Practice of Midwifery.

plained by Dr. William T. Lusk,* who says on this subject: "In these cases the oxytocic effect is probably due to the quieting action exerted upon the spinal nerves. It has been surmised that the nerves of the uterus derived from the cerebro-spinal system possess inhibitory properties, a theory which, if true, readily explains how severe pain suspends uterine action, and how the quieting of pain would restore the motor nerves to their full energy." When an opiate is indicated, McMun's elixir of opium is the preparation that best serves the purpose, for the reason that it is equal to laudanum in its narcotic effect, and does not produce either constipation or nausea. It is to be given in doses of from a half to a teaspoonful, repeated hourly until the desired result is produced.

*The Use of Hydrate of Chlorate.*—From the first introduction of hydrate of chloral into the therapeutics of midwifery, we have used it in our practice with the most gratifying results. This drug in moderate doses is hypnotic and not narcotic in its action, and hence it obtunds the sensibility to pain without stopping the uterine contractions ; it calms nervous excitement, lengthens the intervals between the pains, and thus makes them stronger and more effective. In full doses, hydrate of chloral, like opium, arrests the action of the womb; but its power in this particular is not so great, and is not to be depended upon with any degree of certainty. For the prevention and treatment of puerperal eclampsia, hydrate of chloral is one of the most reliable and valuable of remedial agents; and the routine practice of giving it in the first stage of every case of labor has in our hands been always attended with the good effect of alleviating, to a great extent, the sufferings of the woman, and preventing the occurrence of uterine inertia or atony. This drug may be administered by either the mouth or rectum, as its absorption is equally rapid from the stomach or bowels, and is to be given during labor in doses of fifteen grains every fifteen minutes until sleep is produced, or three doses have been taken. Dr. Robert Barnes† lays special stress upon the point that not more than four

*Science and Art of Midwifery, page 424. †System of Obstetric Medicine and Surgery, page 144.

doses are to be given in this manner, because of the paralyzing action of hydrate of chloral, in large quantities, upon the heart.

*The Use of Chloroform.*—The inhalation of chloroform during the first stage of labor increases the action of the womb by blunting the sensitive spinal centres, and thus abolishing their inhibitory influence. It should be employed, however, with care and caution, and never carried to the extent of complete anæsthesia, for this so relaxes and weakens the uterus as to give rise to serious complications during the second and third stages.

*The Use of Quinine.*—Petitjean,* in 1846, asserted that quinine possessed oxytocic properties and that hence its administration to pregnant women is liable to be followed by abortion or premature labor. This error although contradicted and proven untrue by the experience of all who practice in malarial districts as well as by the physiological experiments of Dr. H. C. Wood and others, is still retained in the books and has yet the stamp of authority.† We have often given quinine in large and repeated doses to women at every period of gestation without in any instance producing the slightest bad consequence, and regard it as one of the very best remedies, in cases of malarial poisoning, for the prevention of miscarriage when threatened and for the relief of false pains due to an hyperesthetic or neuralgic state of the nervous system. This drug in these cases does good, not only as a direct antidote to the miasmatic element, but also because being a powerful nervous sedative it allays spinal irritation. Although quinine has no influence whatever on the pregnant and non-pregnant womb, it certainly when taken in large doses during labor exerts a stimulant action.

Its value in parturition was first announced by Dr. John S. Wilson,‡ in 1855, and has since been confirmed by the observation of other eminent investigators.§ We have found one dose of twenty grains of quinine given in the first stage to be of the greatest benefit, causing the pains to become strong and regular the os to soften and dilate and after delivery firm contraction of the uterine

*Braithwaites Retrospect Vol II. page 222.

†Phillips Materia Medica and Therapeutics, page 255.

‡Southern Medical and Surgical Journal 1855, page 341.

§Treatise on Therapeutics by Trousseau and Pidoux, Vol. III. page 134.

muscle to take place preventing after pains and hemorrhage, cinchonism under these circumstances does not often occur. This mode of administering the drug is advised by Dr. Albert H. Smith,* of Philadelphia, as producing the best results. He believes it acts as a general stimulant and promoter of vital energy and functional activity, and recommends that the bisulphate be used as it is the most soluble of the salts of quinine, and hence more quickly affects the system. Dr. Henry F. Campbell,† has given the most probable explanation of the action of quinine in labor, by calling attention to its virtues as a cerebro spinal depressant. He has shown that the constant recurrence of pain after a time induces a morbid condition of reflex excitement in the nervous system, which while it intensifies the suffering of the woman, effectually retards the progress of the delivery; quinine by obtunding the sensibility of the inhibitory centres of the cord, allows the normal reflexes to go on, and so indirectly increases the action of the uterus. That this drug may be also in some small measure a direct excitant of the hypertrophied muscular faciculi of the parturient uterus, though having no effect upon its undeveloped tissue when in the unimpregnated or early gravid state is made reasonable by the fact that quinine contracts the unstripped fibres of the middle coat of the blood vessels, a property that was first demonstrated by Dr. Robert Campbell,‡ and upon which depends its value in preventing congestion and controlling inflammation.

*The Use of Ergot.*—Powerful oxytocies such as Ergot, cotton root, etc., are never to be employed during the first stage of labor, except as auxiliaries to instrumental or other operative procedures.

*Blood-Letting.*—The use of depressing remedies, namely: venisection, the administration of tarter emetic, ipecac, etc., was in former days much in vogue in the treatment of rigidity of the os and other abnormal conditions met within in first stage of labor. Now a better knowledge of the physiology and pathology of parturition has caused the employment of these agents to be greatly restricted,

*Retarded Dilatation of the Os Uteri, page 27.
†Transactions American Gynecological Society, Vol. V.
‡Southern Medical and Surgical Journal, 1858.

if not almost entirely abandoned. In a very valuable paper, the last that he ever presented to the profession, the late Dr. Samuel D. Gross called attention to the fact that this is often an error, rather than an advance in modern therapeutics, shows the usefulness of blood-letting, tartar emetic, etc., in certain cases, and urges the importance and necessity of a more frequent resort to these remedies in practice. Our own observation has proved to us conclusively the truth of Dr. Gross' views, for we have repeatedly had cases of robust, strong, plethoric women in whom cerebral congestion or eclampsia were imminent, which were perfectly relieved by the moderate abstraction of blood. And we have often seen in these cases a tightly contracted os relax, and become dilated, intense headache disappear and nervous excitement subside. During labor when venisection is required the patient should always be bled while sitting up, so that the system may be quickly impressed by the loss of the smallest quantity of blood. A nice diagnostic perception is necessary in order to determine rightly the cases in which blood-letting is indicated, and a return to the old practice of indiscriminate use of the lancet is very much to be deprecated.

*Artificial Dilatation of the Os Uteri.*—In cases in which the membranes have ruptured before the commencement of, or early in the course of labor, and the liquor amnii has long drained off or in those in which the os, owing to either spastic or organic rigidity or to deficient uterine action, does not open artificial dilation is of great benefit in two ways, viz.: First by mechanically stretching the parts and thus making room for the passage of the child, and second by reflex action stimulating the uterus to more perfect contraction. The finger for obvious reasons is in the majority of instances the best instrument to accomplish this purpose. Digital manipulation to produce dilatation of the os uteri must, however, only be attempted when the cervix is dilatable, that is when it is soft, relaxed and yielding, and should always be done between or during the subsidence of the pains, for the reason that the contractions of the womb begin at the cervix and pass in a wave upward to the fundus, and the os is firmer and smaller at the commencement of a pain, and expands as it is going off, endeavors to effect dilatation at the beginning of a contraction, therefore only adds to the suffering with no other than the bad result of tearing or bruising the muscular tissue. When the amniotic sack is present in practicing digital dilatation, care should be taken to avoid its rupture as long as possible, because we are aided much more in our efforts by the elastic bag of waters than by the hard head of the child, and the premature loss of the amniotic fluid gives rise to all those trying complications comprehended under the term "dry labor" and is especially liable to cause faulty presentations or occipito posterior positions of the vertex. In these cases the finger should be passed high up separating, without breaking the membranes from the wall of the uterus.

When the cervix is undilatable the hydrostatic dilators of Dr. Barnes, of London, are a most valuable resource; they closely imitate the natural process and overcome the resistance of the parts by keeping up a firm, equitable, constant pressure.* It is to be regretted that these bags cannot be constructed of some material that would be more indestructible and that could better stand the wear and tear of practice, for as now made, when once or twice used, they are ruined forever, and if kept without use the india rubber undergoes some chemical change that makes it worthless. Often when required to meet some sudden emergency these bags will be found rotten and bursted and totally unfit for service.

The use of belladonna ointment, atropia and the hot douche against the cervix as a means of promoting relaxation, has proven in our hands of little or no value.

The presence of cicatricial tissue or malignant diseases causing stenosis or occlusion of the os sometimes makes the use of the knife necessary. A remarkable case of this kind occurred in our practice, and is reported in the transactions of the American Gynecological Society for 1880. The occlusion in this case was complete and was due to adhesive inflammation following the application of the cyanide of mercury to the cervix during pregnancy for the cure of obstinate ulceration; the operation of vaginal

*Obstetric Operations, page 102.

hysterotomy had to be performed before delivery could be accomplished. Obliquity of the uterus and malpositions of its neck may sometimes be mistaken for occlusion; examination in these cases usually shows the head low in the pelvis covered by the streached anterior wall of the womb and the os closed and turned high up against the promintory of the sacrum; dilatation is readily effected by changing the position of the woman, the adjustment of an abdominal bandage and by means of the finger, drawing down the cervix into the axis of the superior strait so that the pressure of the amniotic sack and the presenting part can bear properly upon it. The knife ought to be employed in stenosis and occlusion, only after all other means have failed to effect dilatation and never in cases of simple rigidity, because its use is very apt to be followed by acute inflammation and the incisions made offer a ready way for the entrance of septic matters into the blood.

*The Use of the Forceps.*—In 1863 Dr. Isaac E. Taylor first suggested and practiced the early use of the forceps in the first stage of natural labor. This was then a decided innovation in practice and totally at variance with all recognized obstetric authority and has not as yet received the full assent and acceptance of the profession. As we have had occasion many times to resort to this operation, and have seen its great value in certain cases, we desire here to give the results of our experience and to add our testimony to that of others as to its practicability, safety and life-saving usefulness.

During the first stage of labor neither traction, compression nor leverage is to be made by the forceps in the same manner as when these instruments are applied in the second stage. They are to be employed, not to forcibly or rapidly pull the head through the cervix, but simply to hold it properly in contact with the internal os during a pain, and thus aid the natural powers in effecting dilatation. When used in this way the forceps promote flexion, and by their presence within the uterus excite more regular and perfect contraction of that organ. This reflex or so-called dynamic action is best exerted when the patient is not in a state of anæsthesia, and hence, whenever possible, these instruments should be applied without chloroform during the first stage of labor. There is no especial danger in the introduction of the forceps into the interior of the womb, nor is the risk proportionate to the size of the os at the time of the operation, for often when used in the second stage the blades have to be passed high up within the uterine cavity without any attendant bad results, and clinical experience has shown that by their proper use no more harm is done in the first stage through an undilated os than in the second stage through a contracted vulval orifice. Dr. Taylor* has proved both by examination of many living patients, and by close study of *post-mortem* specimens that lacerations of the cervix in such cases very seldom occur, and Dr. George Johnson,† from statistics of one hundred and sixty-nine cases under his charge in the Rotunda Lying-in Hospital of Dublin, in which the forceps were applied before the os uteri was fully open, says "the practice is not alone safe and justifiable, but also a great preservative of the lives of both mothers and children."

It is to be fully understood that it is the *early* and not the *frequent* use of the forceps during the first stage of labor that is advised and advocated, for cases are not often met with in which a resort to this procedure is proper or necessary. In the Report of the Rotunda Hospital for 1876, Dr. Johnson very truly says on this subject: "This practice should never be adopted merely in order to save our own time, nor should it be attempted when the os uteri is rigid; in such cases the proper means should be taken to overcome this state, and it should alone be had recourse to when the life of the mother or that of her offspring is in jeopardy." This correctly expresses what the indications are for the use of the forceps in the first stage of natural labor, and it is an ignorance or misunderstanding of these, and a failure to appreciate its true scope, and the ends to be attained by it that has led to the untimely and improper performance of this operation, and to all the evils resulting therefrom ; and it is this abuse that has caused the great opposition to it on the part of many distinguished obstetricians.

---

*The Early Use of the Forceps in the First Stage of Natural Labor. By Isaac E. Taylor, M. D.

†Obstetric Journal of Great Britain and Ireland, March, 1879.

Taylor's obstetric forceps, devised for use early in the first stage, are chiefly peculiar in having very long narrow blades, so as to make their introduction possible through a very contracted cervix, and a lock with a spring attached, by means of which compression of the head is made just sufficient to cause the instrument to be self-retaining. These forceps can be passed without damage being done through an os measuring only an inch in diameter, and when used, nature is to be followed as much as possible; thus by them the occiput should be brought down at the beginning of a pain and held during its continuance, and for a short time after it has subsided, and then without removing the instrument be allowed to recede in the interval between the uterine contractions. Contact of the head with the cervix should never be constant, because by this the maternal parts are much injured, and the child unduly bruised and compressed. These manipulations must be practiced with the utmost care and patience, and therefore cases of this kind often require two or three hours to complete the dilatation of the os and effect the delivery. Dr. Taylor advises that after the os has been fully opened and the head drawn down into the pelvis, that his forceps be removed and nature be allowed to finish the delivery, or a pair of short forceps be applied. This, however, we have never done, believing that while the instruments are in position it is best by them to terminate the labor.

In concluding our remarks on this subject we would say, that since modern research has increased our knowledge of certain old remedies and given us the priceless new ones, chloroform and hydrate of chloral, and inventive genius has devised new operative procedures, and extended the application of the old, nothing in the whole range of obstetric medicine is now so certain and satisfactory as the treatment of delay in the first stage of natural labor.

